# Exploring self-rated health, physical activity, and social anxiety among female Chinese university students: a variable- and person-centered analysis

**DOI:** 10.3389/fpubh.2025.1681504

**Published:** 2025-09-18

**Authors:** Wenying Huang, Bin Chen, Chang Hu

**Affiliations:** Physical Education College, Jiangxi Normal University, Nanchang, China

**Keywords:** self-rated health, physical activity, social anxiety, female college students, mediation analysis, latent profile analysis

## Abstract

**Background:**

Physical inactivity is a significant public health issue among female college students. This study aimed to explore the complex relationship between self-rated health (SRH), social anxiety (SA), and physical activity (PA) in a sample of female Chinese university students, employing both variable-centered and person-centered approaches.

**Methods:**

A cross-sectional survey was conducted with a sample of 1,452 female Chinese university students who completed the Self-Perceived Health Scale (to measure SRH), the Social Interaction Anxiety Scale–6 and the Social Phobia Scale–6 (to measure SA), and a validated single-item measure of PA (to measure PA). First, a mediation model was used to test the indirect effect of SRH on PA through SA. Second, Latent Profile Analysis was conducted to identify distinct subgroups based on individuals’ SRH and SA levels. Finally, ANOVA was used to examine PA differences across the identified profiles.

**Results:**

The mediation analysis revealed that SA partially mediated the relationship between SRH and PA, accounting for 12% of the total effect (CI [0.011, 0.090]). The Latent Profile Analysis identified three distinct profiles: “Healthy-Resilient” (21.62%), characterized by high SRH and low SA; “Moderate-Adapting” (70.39%), with average SRH and SA; and “Unhealthy-Anxious” (7.99%), with low SRH and high SA. The “Unhealthy-Anxious” group reported significantly lower levels of PA than the other two groups.

**Conclusion:**

Our findings suggest that SA is a significant psychological mechanism linking SRH to PA among female Chinese university students. From a public health perspective, these findings highlight the importance of addressing mental health factors, such as SA, in interventions designed to promote PA in this population. The identification of distinct subgroups underscores the need for tailored interventions over one-size-fits-all approaches.

## 1 Introduction

Physical activity (PA) is any bodily movement produced by skeletal muscles that results in energy expenditure (1–4). It is widely recognized that regular PA is a cornerstone of maintaining physical and mental health and preventing chronic diseases (5–7). The university period is a critical life stage for the formation of personal habits, and PA patterns established during this time have a decisive impact on an individual’s long-term health trajectory (8, 9). Despite its benefits, insufficient PA among female university students remains a global health concern (10–12). The World Health Organization has consistently highlighted a gender disparity in activity levels, noting that women are generally less active than men (13, 14). This phenomenon is especially evident among adolescents and young adults (5, 15). For example, a large-scale study involving female university students found that nearly 35% were sedentary, with another 34% engaging in PA only sporadically (16). Similarly, other research indicates that a substantial portion of female students fail to meet the recommended guidelines of 150 min of moderate-to-vigorous PA per week (17). Female university students in China may be a particularly vulnerable population for physical inactivity (18, 19). They often face a unique confluence of stressors, including intense academic competition and societal expectations related to traditional gender roles, which may prioritize academic achievement over physical wellbeing (20, 21). This, combined with the newfound independence and lifestyle changes that accompany university life, can make it challenging for young women to establish and maintain healthy physical activity habits (22, 23). Therefore, identifying and understanding the psychological factors that influence PA participation in this specific population is crucial for developing effective interventions.

In addition, among the numerous potential factors, Self-Rated Health (SRH), an individual’s subjective perception and overall evaluation of their own health status, has garnered considerable academic attention (24–26). While objective health indicators are valuable, SRH provides a more holistic and personally meaningful assessment of an individual’s overall health state (27). It captures a person’s subjective experience of their health, which is a powerful predictor of health behaviors, morbidity, and mortality, even after controlling for objective health status (28). In the context of our study, SRH is particularly relevant as we are interested in how an individual’s perception of their health influences their engagement in health-promoting behaviors like physical activity.

## 2 Literature review and hypotheses development

### 2.1 Self-rated health and physical activity

SRH is not an objective physiological measure, yet it has been proven to be a robust predictor of future health outcomes and mortality across diverse populations (29, 30). Recent research continues to reinforce this connection, demonstrating a consistent positive association between higher SRH and greater engagement in health-promoting behaviors, including PA (31–34). For instance, a large cross-sectional survey of adolescents found a strong, positive dose–response relationship, where more frequent vigorous PA was linked to better SRH (35). Similarly, a 2025 study underscored that SRH remains a key moderating factor, where the protective effects of PA on mental health outcomes were significantly more pronounced in individuals who rated their health as good (36). This growing body of evidence suggests that an individual’s subjective health belief is a critical cognitive antecedent to action (37). Nevertheless, this association’s psychological pathway remains under-investigated, particularly among young adults. In other words, how an individual’s “feeling” about their health translates into “actual” PA behavior is a process that warrants deeper investigation.

### 2.2 The mediating role of social anxiety

This study posits that Social Anxiety (SA) may play a critical mediating role in the relationship between SRH and PA. SA is defined as a marked fear or anxiety in social or performance situations where one might be scrutinized by others (38, 39). Given that many forms of PA (e.g., gym workouts, team sports) take place in public or semi-public settings, they inherently constitute potential social performance contexts (40). According to self-presentation theory, individuals with a lower evaluation of their own state (such as health or physical appearance) are more likely to anticipate negative evaluation in social settings, thereby triggering anxiety (41–43). The influence of SA on physical activity may be particularly pronounced in collectivist cultures, such as China (44). In these cultural contexts, where there is a strong emphasis on social harmony and avoiding loss of face, the fear of being negatively evaluated by others can be a powerful deterrent to participating in public activities, including sports and exercise. This may be especially true for women, who may face greater scrutiny regarding their physical appearance and competence in physical activities. It follows logically that lower SRH could lead to avoidance of PA by heightening SA, particularly anxiety related to one’s physical appearance, as a strategy to evade potential social discomfort (45, 46). Thus, SA is a likely psychological bridge connecting poor health perception to lower activity levels, and verifying this mediation pathway is the primary objective of this study.

### 2.3 The variable-centered and person-centered approaches

Previous research on these variables has predominantly relied on “variable-centered” approaches (e.g., structural equation modeling), which aim to estimate the average relationships among variables for an entire sample (47, 48). A fundamental limitation of this approach is its “population-average” assumption, which presumes that all individuals follow the same psychological model (49). This assumption may obscure significant heterogeneity within the population. For instance, not all individuals with low SRH will experience the same degree of SA, and the resulting impact on their PA may also differ. Overlooking such potential heterogeneity limits a comprehensive understanding of the phenomenon and may lead to inadequately targeted intervention strategies.

To address this methodological gap, this study adopts a complementary “person-centered” strategy, namely Latent Profile Analysis (LPA) (50, 51). Unlike variable-centered methods that examine relationships between variables, LPA aims to identify data-driven, qualitatively distinct latent subgroups based on individuals’ response patterns across a set of observed variables (52). This approach allows us to investigate a more profound question: Do specific subgroups, defined by unique combinations of SRH and SA levels, exist among female university students? If so, do their PA patterns also exhibit significant differences? By identifying these potential “health-anxiety” profiles, we can move beyond a focus on “average effects” and toward an analysis of the specific risk patterns of different profiles of individuals.

### 2.4 Aims and objectives of the study

In summary, this study employs a dual-analytic approach, integrating both variable-centered and person-centered methods, to comprehensively investigate the relationships among SRH, SA, and PA in female university students. The research will first test the mediating effect of SA using structural equation modeling to uncover the average causal pathway. Subsequently, it will utilize LPA to identify subgroups with distinct “health-anxiety” characteristics and examine the differences in PA levels across these groups. By integrating the findings from both methods, this study aims to provide new insights into the complex psychological mechanisms influencing PA among female university students and to offer empirical support for the future development of personalized intervention programs based on the characteristics of different subpopulations. Based on the theoretical framework and literature review presented above, this study proposes the following hypotheses: H1 (SA will mediate the relationship between SRH and PA); H2 (There will be distinct and meaningful latent profiles of female college students based on their levels of SRH and SA); H3 (These profiles will differ significantly in their levels of PA).

## 3 Materials and methods

### 3.1 Participants and procedure

This study adopted a cross-sectional survey design, conducted through an online questionnaire platform. To ensure sufficient statistical power for our analyses, an *a priori* power calculation was performed using G*Power 3.1 software. For a standard regression-based mediation model (53), with a desired power (1 − *β*) of 0.95 and a significance level (*α*) set at 0.05, a minimum of 119 participants would be required to detect a medium effect size (*f*^2^ = 0.15). The final sample size obtained in this study substantially surpasses this calculated threshold, providing robust statistical power for the subsequent analyses.

This study utilized a convenience sampling strategy for recruitment from six universities across Jiangxi and Yunnan provinces in China. Data collection over a one-month period beginning March 17, 2025. To be included in the study, individuals had to meet the following criteria: (a) Participants must be biologically female; (b) be currently enrolled as a university student; and (c) provide informed consent to participate. Exclusion criteria were established to ensure data quality and sample homogeneity, and included: (a) a self-reported history of any formally diagnosed mental health disorder; (b) failure to complete the entire survey; or (c) responses flagged as invalid due to excessively short completion times, which would suggest inattentive participation. In addition, to ensure data reliability, missing data and outliers were excluded from the study. An initial pool of 1,538 individuals accessed and responded to the survey. Following the application of the pre-defined inclusion and exclusion criteria, 86 responses were removed from the dataset. This screening process resulted in a final valid sample of 1,452 participants. The age of the participants in the final sample ranged from 18 to 30 years.

Informed consent was obtained from all participants before they began the survey. The consent form clearly outlined the study’s objectives, the voluntary nature of participation, data confidentiality measures, and the participant’s right to withdraw at any point without penalty. As a token of appreciation for their time and effort, each individual who submitted a valid and complete survey received compensation of 3 CNY (US$0.42). All study procedures received ethical approval from our institution (IRB-JXNU-PEC-2025019).

### 3.2 Measures

#### 3.2.1 Self-rated health

Participants’ SRH was evaluated using the Self-Perceived Health Scale (SPHS-12) developed by Tinajero-Chávez et al. (54). This instrument comprises 12 items distributed across three sub-dimensions: physical health, psychological health, and healthy lifestyle. Respondents indicated their level of agreement with each statement (e.g., “I can keep my balance, for example, by standing on one foot”) on a 6-point Likert scale, anchored at 1 (Disagree) and 6 (Absolutely agree). The SPHS-12 yields a raw total score ranging from 12 to 72. To facilitate interpretation, we divided the total score by the number of items (12) to obtain a mean score ranging from 1 to 6. The mean score of 3.666 reported in the results therefore reflects the average item score rather than the raw total score. Higher mean values indicate better self-rated health. In the current study, the Cronbach’s *α* of 0.890 indicates good internal consistency.

#### 3.2.2 Physical activity

A single-item measure with established validity in large-scale research was employed to assess PA. Participants were asked to respond to the question: “Over the past 7 days, how many days did you engage in at least 20 min of physical exercise or other activity that made you sweat or breathe heavily?.” Responses, ranging from 0 to 7 days, were treated as a continuous variable for statistical analysis. Although this method cannot fully represent the participants’ level of PA, it has shown good effectiveness in several large-scale studies conducted previously (55–57).

#### 3.2.3 Social anxiety

SA was assessed using two widely recognized and complementary short-form scales: the Social Interaction Anxiety Scale (SIAS-6) and the Social Phobia Scale (SPS-6) (58). Each scale contains six items. Participants rated how characteristic a statement was of them using a 5-point scale (0 = “Not at all characteristic of me” to 4 = “Extremely characteristic of me”). Higher mean scores on each scale indicate more severe symptoms. Both instruments have demonstrated strong psychometric properties and have been extensively validated for use with Chinese university students (59–61). For the current sample, good internal consistency was confirmed, with a Cronbach’s *α* of 0.829 for the SIAS-6 and 0.799 for the SPS-6.

### 3.3 Data analysis

The data were processed and analyzed using SPSS 26.0 and Mplus 8.3, with the threshold for statistical significance established at *p* < 0.05 for all tests (62). Initially, preliminary analyses were conducted, including the calculation of descriptive statistics and Pearson correlation coefficients to ascertain the basic characteristics and interrelationships of the key study variables. Subsequently, the variable-centered analysis proceeded by testing the proposed mediation pathway. Specifically, the PROCESS macro (Model 4) for SPSS was employed to investigate the indirect effect of SRH on PA via the mediating variable of SA. To rigorously assess the significance of this indirect effect, a bootstrapping procedure involving 5,000 resamples was executed to generate 95% bias-corrected confidence intervals, with an effect considered significant if its Confidence interval did not encompass zero.

To complement the variable-centered findings, a person-centered approach was implemented using LPA within Mplus 8.3. A systematic process of model comparison was undertaken, beginning with a one-profile solution and incrementally adding profiles. The selection of the optimal model was a multifaceted decision, guided by a comprehensive evaluation of statistical fit indices, including the Akaike Information Criterion (AIC), Bayesian Information Criterion (BIC), and the sample-size adjusted BIC (aBIC) (63). Further consideration was given to the Lo–Mendell–Rubin and Bootstrapped Likelihood Ratio Tests (LMR-LRT and BLRT) to determine if a model with more profiles offered a statistically significant improvement in fit. The clarity of classification, as indicated by the Entropy value, along with the theoretical coherence and parsimony of the resulting profiles, were also critical factors in the final determination. Once the optimal profile solution was identified, a one-way ANOVA, followed by Bonferroni post-hoc tests for pairwise comparisons, was conducted to determine if profile membership was associated with significant mean differences in PA.

## 4 Results

### 4.1 Demographic information on female college students

Table 1 summarizes the demographic characteristics of the sample, including education level, place of birth, ethnicity, and BMI categories. A one-way ANOVA revealed a highly significant effect of education on SA scores (*F* = 5.674, *p* < 0.01) and also showed significant effects of BMI on both SRH (*F* = 4.168, *p* < 0.01) and SA (*F* = 3.501, *p* < 0.05). The independent samples t-test indicated that there were significant differences between ethnic groups for PA (*t* = 2.025, *p* < 0.05) and SA (*t* = −2.619, *p* < 0.01). In contrast, no significant differences were found based on place of birth for any of the three outcome variables.

**Table 1:** Basic demographic characteristics of participants.

	*N* (%)	SRH	PA	SA
Education		1.455	1.155	5.674^**^
Undergraduates	1,124 (77.41%)			
Master’s students	291 (20.04%)			
Doctoral students	37 (2.55%)			
Place of birth		−1.931	−0.063	−0.019
City	797 (54.90%)			
Country	655 (45.10%)			
Ethnicity		1.313	2.025^*^	−2.619^**^
Han	1,291 (88.91%)			
Minority	161 (11.09%)			
BMI		4.168^**^	0.149	3.501^*^
<18.5	364 (25.07%)			
18.5–23.9	509 (35.05%)			
24–27.9	424 (29.20%)			
>28	155 (10.68%)			

Independent Samples t-test and ANOVA results (two-tailed test). *p < 0.05, **p < 0.01. SRH, Self-Rated Health; PA, Physical Activity; SA, Social Anxiety.

### 4.2 Correlation analysis

The descriptive statistics and Pearson correlation coefficients for the primary study variables indicated that SRH was significantly and positively correlated with PA, suggesting that students who perceived their health more favorably reported higher levels of PA. Conversely, SRH was strongly and negatively correlated with SA. Furthermore, a significant negative correlation was observed between PA and SA, indicating that higher SA was associated with less engagement in PA (Table 2).

**Table 2:** Correlation coefficients of self-rated healthy, social anxiety, and physical activity in female college students.

	*M*	*SD*	Skewness	Kurtosis	SRH	PA	SA
SRH	3.666	1.088	0.214	−0.379	1		
PA	4.097	2.009	−0.123	−1.168	0.411^**^	1	
SA	1.570	0.827	0.061	−0.908	−0.623^**^	−0.306^**^	1

**p < 0.01 (two-tailed).

### 4.3 The mediation analyses

**Figure 1:**
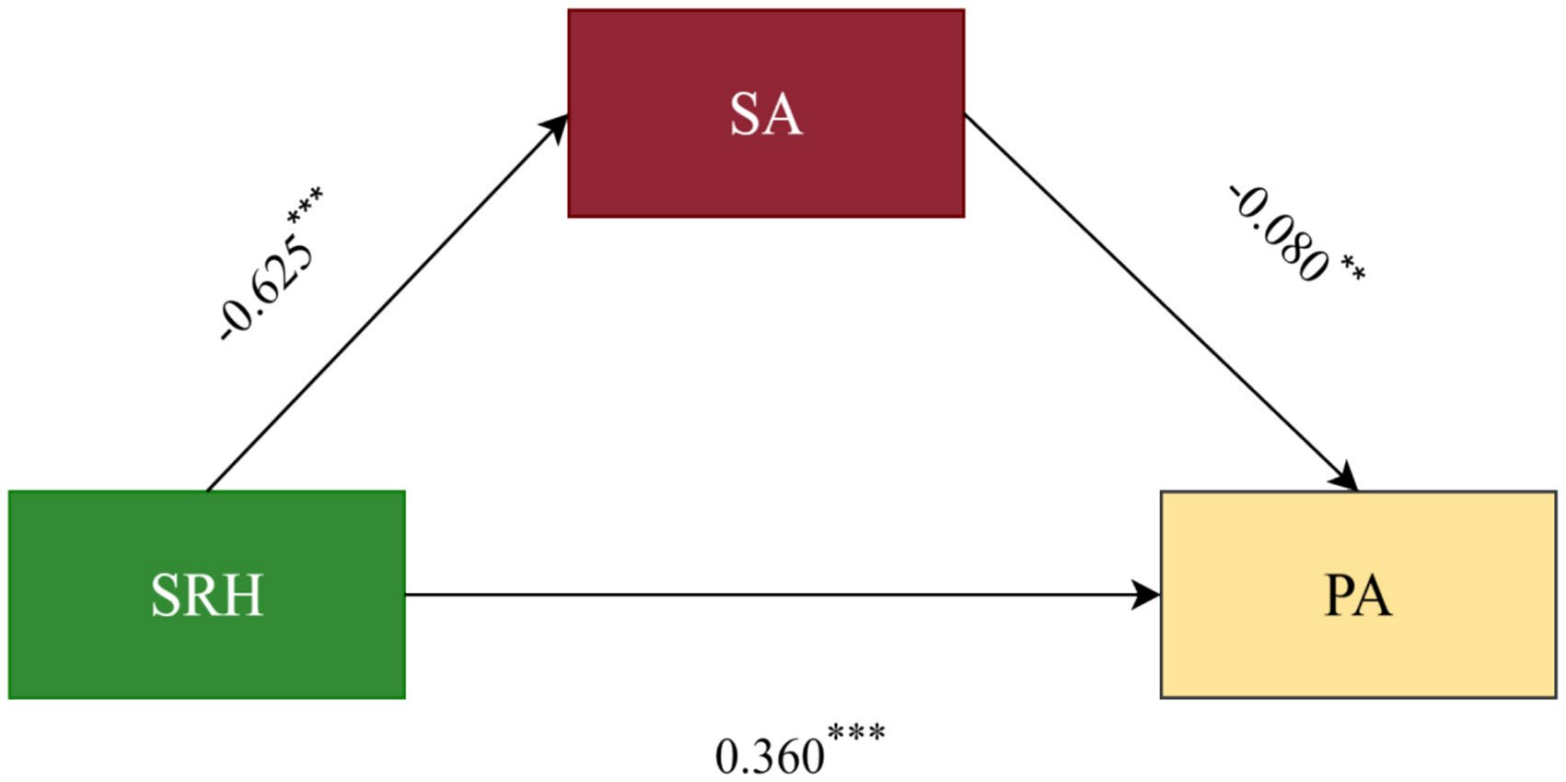
Mediation effect diagram. The effect of SRH on the mediator, SA, was significant (*β* = −0.625, *p* < 0.001). The effect of the mediator, SA, on PA was also significant (*β* = −0.080, *p* < 0.01). The direct effect of SRH on PA remained significant (*β* = 0.360, *p* < 0.001).

To test Hypothesis 1, we examined the mediating role of SA in the relationship between SRH and PA using the PROCESS macro (Model 4). First, a collinearity diagnosis was conducted. The results showed that the Variance Inflation Factor for the variables was 1.636, well below the common threshold of 10, indicating that multicollinearity was not a concern. For the mediation analysis, we controlled for demographic variables as potential confounding variables (Table 3). These covariates were selected based on preliminary analyses and their established association with health and physical activity in the literature. The effect of SRH on the mediator, SA, was significant (*β* = −0.625, *p* < 0.001). The effect of the mediator, SA, on PA was also significant (*β* = −0.080, *p* < 0.01). The direct effect of SRH on PA remained significant (*β* = 0.360, *p* < 0.001) (Figure 1). Crucially, the indirect effect of SRH on PA through SA was also statistically significant (*β* = 0.050, Boot se = 0.020), with a 95% bias-corrected confidence interval that did not contain zero [0.011, 0.090] (Table 4). This result supports H1, indicating that SA partially mediates the relationship between SRH and PA. The mediating effect accounted for 12% of the total effect.

**Table 3:** Mediation effect regression results.

Variables	Model 1		Model 2		Model 3	
	(SA)		(PA)		(PA)	
	*β*	*t*	*β*	*t*	*β*	*t*
SRH	−0.625^***^	−30.452	0.360^***^	11.734	0.410^***^	17.087
SA			−0.080^**^	−2.605		
Education	0.143	1.902	0.094	1.074	0.083	0.943
Ethnicity	0.161^*^	2.462	−0.113	−1.481	−0.126	−1.650
BMI	−0.020	−0.913	−0.004	−0.160	−0.002	−0.097
Place of birth	0.066	1.604	−0.034	−0.713	−0.040	−0.822
Age	−0.021	−1.220	−0.010	−0.525	−0.009	−0.441
*R* ^2^	0.394		0.176		0.172	
*F*	156.652^***^		44.007^***^		50.001^***^	

^*^p < 0.05, ^**^p < 0.01, ^***^p < 0.001.

**Table 4:** Mediating effects of social anxiety between self-rated healthy and physical activity.

	Effect	Boost se	95%CI		Relativistic effect
			LLCI	ULCI	
Total	0.410	0.024	0.363	0.457	
SRH → PA	0.360	0.031	0.300	0.420	88%
SRH → SA → PA	0.050	0.020	0.011	0.090	12%

### 4.4 Latent profile analyses

**Figure 2:**
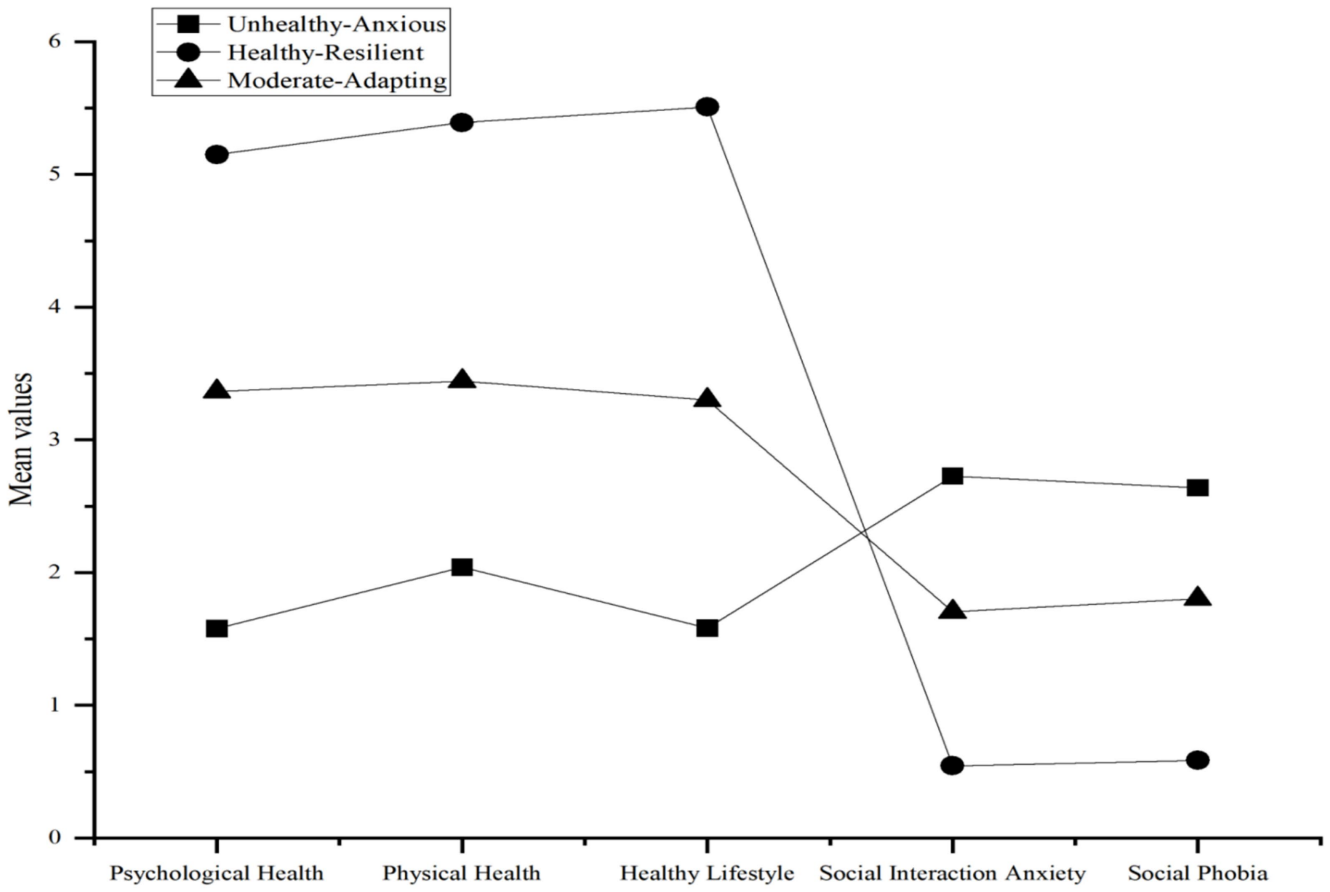
Latent profiles of self-rated health and social anxiety.

In line with Hypothesis 2, we conducted an LPA to identify distinct subgroups of female college students based on their levels of SRH and SA. We compared models with one to five latent profiles. As shown in the LPA table, the fit indices (AIC, BIC, and aBIC) continued to decrease as more profiles were added (Table 5). However, considering the principle of parsimony, theoretical interpretability, and the significant LMR and BLRT tests (*p* < 0.001), the three-profile solution was selected as the optimal model. This solution also demonstrated excellent classification accuracy, with a high entropy value of 0.971. The three identified profiles were named based on their distinct patterns of SRH and SA: Unhealthy-Anxious (A1, *n* = 116, 7.99%). This group, the smallest of the three, was characterized by the lowest SRH and the highest levels of SA (Figure 2). Healthy-Resilient (A2, *n* = 314, 21.62%): This group was characterized by the highest levels of SRH and the lowest levels of SA. Moderate-Adapting (A3, *n* = 1,022, 70.39%): This was the largest group, representing the majority of students, who reported average levels of both SRH and SA.

**Table 5:** Comparison of fit indices for latent profile analysis models.

Model	AIC	BIC	aBIC	LMR(*p*)	BLRT(*p*)	Entropy	Categorical probability%
Class 1	22,393.21	22,446.02	22,414.25				
Class 2	19,829.73	19,914.22	19,863.40	<0.0001	<0.0001	0.972	78.10%/21.90%
Class 3	18,950.21	19,066.38	18,996.50	<0.0001	<0.0001	0.971	7.99%/
							21.62%/70.39%
Class 4	18,501.60	18,649.46	18,560.51	<0.0001	<0.0001	0.924	52.48%/7.64%/
							18.39%/21.49%
Class 5	18,290.68	18,470.23	18,362.22	<0.0001	<0.0001	0.845	7.92%/
							17.91%/31.47%/
							21.28%/21.42%

### 4.5 Effect of latent profile classification

To test Hypothesis 3, a one-way ANOVA was conducted to examine whether physical activity levels differed significantly across the three latent profiles. The analysis revealed a significant main effect of profile membership on PA, *F* (2, 1,449) = 167.276, *p* < 0.001, *η*^2^ = 0.118 (Table 6).

**Table 6:** Group differences in PA across latent profiles.

Variable	A1 (116)	A2 (314)	A3 (1,022)	*η* ^2^	*F*
PA	2.431 ± 1.669	5.592 ± 1.093	3.827 ± 1.995	0.118	167.276^***^

***p < 0.001. A1 (Unhealthy-Anxious), A2 (Healthy-Resilient), A3 (Moderate-Adapting).

Subsequent post-hoc comparisons employing the Bonferroni test revealed significant disparities among all three groups. Specifically, the “Healthy-Resilient” group (*M* = 5.59, *SD* = 1.09) reported significantly more physical activity than both the “Moderate-Adapting” group (*M* = 3.83, *SD* = 2.00) and the Low “Unhealthy-Anxious” group (*M* = 2.43, *SD* = 1.67). Furthermore, the “Moderate-Adapting” group engaged in significantly more physical activity than the “Unhealthy-Anxious” group. These findings fully support H3, demonstrating that the identified health-anxiety profiles are associated with distinct patterns of physical activity behavior (A2 > A3 > A1) (Table 7).

**Table 7:** Multiple comparisons result.

Dependent Variable: PA	(I) Class	(J) Class	Mean difference (I–J)	STD. Error	Sig.	95% CI		Outcome
Bonferroni						Lower Bound	Upper Bound	
	A1	A2	−3.161^*^	0.197	<0.001	−3.633	−2.689		A3	−1.396^*^	0.178	<0.001	−1.821	−0.970		A2	A1	3.161^*^	0.197	<0.001	2.689	3.633		A3	1.766^*^	0.117	<0.001	1.485	2.046		A3	A1	1.396^*^	0.178	<0.001	0.970	1.821		A2	−1.766^*^	0.117	<0.001	−2.046	−1.485									A2 > A3 > A1

*p < 0.05.

## 5 Discussion

The present study provides novel insights into the interplay of self-perceived health, social anxiety, and physical activity among female Chinese university students. It is important to interpret these findings within this specific demographic and cultural context, as the observed relationships and profiles may differ in other populations. The variable-centered mediation analysis revealed how SRH relates to PA, identifying SA as a significant psychological mechanism. Poorer self-perceived health is associated with higher SA, which in turn is linked to lower physical activity. The person-centered analysis complements this by identifying who is most affected. The “Unhealthy-Anxious” profile represents a subgroup for whom this mediating pathway is likely most pronounced. This group, characterized by the combination of low SRH and high SA, exhibited the lowest levels of PA.

### 5.1 The mediating effect of social anxiety

A core finding of this study is that SA plays a significant partial mediating role in the relationship between SRH and PA. This result not only statistically validates our hypothesis but, more importantly, reveals a specific psychological pathway. Specifically, when a female student perceives herself as “unhealthy,” this negative self-cognition can become a psychological vulnerability (64), making her more likely to anticipate scrutiny and negative evaluation in settings that require physical “display,” which in turn triggers or exacerbates her SA (65–67). This finding is highly consistent with the tenets of self-presentation theory and resonates with a large body of existing research (68). For example, prior studies have confirmed that lower SRH is associated with greater negative emotional experiences such as depression and anxiety (69–72); our study further links this psychological vulnerability to SA in specific contexts. Concurrently, the role of SA as a potent inhibitor of PA participation has been well-documented (73). The distinctive contribution of our research lies in the integration of these three variables into a unified model, thereby elucidating the chain of effects from “health perception” to “SA” to “activity behavior.” This pathway also can be understood through a Cognitive-Behavioral theory (74). A low self-rating of health acts as a negative core belief about one’s physical self. According to Cognitive-Behavioral theory, when an individual with this belief enters a social-evaluative context, such as a gym or group sport, this cognition is activated (75). This triggers a cascade of anxious thoughts (e.g., “Others will notice how unfit I am”) and a hypervigilance to physiological sensations (e.g., sweating, heavy breathing), which are catastrophically misinterpreted as signs of incompetence or physical failure. The resulting social anxiety then motivates avoidance behaviors—in this case, avoiding physical activity—to prevent the feared social judgment. Thus, SA is not merely correlated with SRH and PA; it serves as the cognitive and emotional bridge that translates a negative health perception into a behavioral pattern of inactivity. The practical implication of this finding is that it shifts the focus of intervention from a relatively vague and difficult-to-change variable (SRH) to a more specific and actionable psychological target (SA). These findings are particularly resonant within the Chinese cultural context. Factors such as the emphasis on academic performance over physical pursuits and Confucian-heritage values that emphasize group harmony and “saving face” can heighten social-evaluative fears (76), potentially making SA a more potent barrier to PA than in Western cultures (77, 78). This may be especially salient for female students, who often face distinct societal pressures regarding physical appearance and competence in traditionally male-dominated domains like sports (79). The fear of failing to meet these aesthetic and performance standards can compound the anxiety, making public physical activity a high-stakes event (80). Furthermore, a cultural reluctance to openly discuss mental health may lead students to somaticize their anxiety, focusing on perceived physical health deficits (low SRH) rather than acknowledging the underlying social fear (71, 81, 82). Therefore, to effectively promote PA among female students with low SRH, providing health education or emphasizing the benefits of exercise is insufficient; it is imperative to concurrently address the underlying SA.

### 5.2 Latent profile of self-rated health and social anxiety

Consistent with Hypothesis 2, this study successfully employed a person-centered approach to identify three distinct latent profiles among female college students based on their SRH and SA. This finding is crucial as it moves beyond a variable-centered understanding to reveal the significant heterogeneity within this population. The three profiles were named “Healthy-Resilient,” “Moderate-Adapting,” and “Unhealthy-Anxious.”

The “Moderate-Adapting” group was the largest, encompassing over 70% of the students. This profile represents the normative experience for female college students, who are typically navigating a complex environment characterized by moderate academic and social pressures (83, 84). Their average levels of SRH and SA likely reflect a general adaptation to the daily stressors of university life, representing a functional equilibrium (85). While this group may not be at immediate risk, they could be susceptible to shifting toward a more maladaptive profile if stressors increase without adequate coping resources.

In stark contrast, the “Healthy-Resilient” group (21.62%) represents a highly adaptive group. These students exhibit a synergy of positive psychological attributes. Their high SRH likely reflects not only good physical health but also a robust sense of self-efficacy and a positive self-concept (86). This psychological fortitude acts as a buffer against social stressors (87). Consequently, their low SA is a logical correlate; with strong self-confidence and positive social experiences, they are less likely to fear negative evaluation from others. This profile aligns with literature on positive psychology and resilience, embodying the ideal state that health promotion initiatives often aim to cultivate (88–90).

Most critically, the analysis identified an “Unhealthy-Anxious” group. Although the smallest group (7.99%), its existence is of significant clinical and practical importance. This profile represents a maladaptive state where poor health perception and intense social fear appear to be mutually reinforcing. The low SRH could stem from objective health problems, but it is equally likely to be magnified by a negative cognitive bias, where individuals are prone to catastrophize minor physical symptoms (91, 92). Simultaneously, high SA, particularly the fear of negative evaluation, can lead to hypervigilance toward one’s own body and its functions, causing any perceived flaw or physiological response to be interpreted as a sign of poor health (93–95). This creates a debilitating vicious cycle: feeling unhealthy heightens social self-consciousness, and fearing social judgment amplifies the negative perception of one’s health. This group is likely to experience significant impairments in their social and academic functioning, extending far beyond the context of physical activity.

While person-centered studies combining SRH and SA are nascent, the “Unhealthy-Anxious” profile is conceptually consistent with high-risk typologies identified in variable-centered research among Western youth, which often link poor health perceptions with internalizing symptoms like anxiety and depression (96, 97). However, the cultural emphasis on social harmony and avoidance of “losing face” in China may make this specific combination of poor health perception and social fear a particularly potent driver of inactivity compared to individualistic cultures (82). Future cross-cultural research is needed to verify if these exact profiles emerge in other populations.

### 5.3 Effect of latent profile classification on physical activity

Supporting Hypothesis 3, the one-way ANOVA results confirmed that the three identified profiles correspond to significantly different levels of physical activity, following a clear pattern: the “Healthy-Resilient” group was most active, followed by the “Moderate-Adapting” group, with the “Unhealthy-Anxious” group being the least active. This finding powerfully validates the person-centered approach by linking distinct psychological typologies to tangible behavioral outcomes. The high activity level of the “Healthy-Resilient” group is well explained by Self-Determination Theory, a motivational framework that posits human behavior is driven by the basic psychological needs for autonomy, competence, and relatedness (98). According to this theory, behaviors are sustained when these needs are satisfied (99, 100). This group likely engages in physical activity out of intrinsic motivation, finding it enjoyable and personally rewarding (101). Their high SRH reflects a strong sense of competence, while their low SA allows them to feel relatedness in group settings without fear, thus creating a self-reinforcing, positive cycle of engagement.

The “Moderate-Adapting” group, representing the majority, displayed an intermediate level of physical activity. Their behavioral patterns can be understood through the lens of the Transtheoretical Model of Health Behavior Change: a stage-based model describing how individuals progress through precontemplation, contemplation, preparation, action, and maintenance in adopting health behaviors (102). This group is likely not in a stable state of maintenance but rather in the “Contemplation” or “Preparation” stages, weighing the pros and cons of regular exercise (103). Their engagement is therefore more variable and highly susceptible to situational factors. Lacking the robust intrinsic motivation of the resilient group and the significant psychological barriers of the anxious group, their decision to be active may depend heavily on external influences (104). Positive factors like social support from peers, accessible and appealing campus facilities, or a manageable academic workload can serve as catalysts, moving them toward the “Action” stage (105). Conversely, negative stressors like exam periods or social conflicts can easily tip the decisional balance, leading to relapse or sustained inactivity. This makes them a crucial target for broad-based, low-threshold university wellness programs designed to provide the necessary “nudge” to foster consistency.

Conversely, the profound inactivity of the “Unhealthy-Anxious” group is best explained by Cognitive-Behavioral Models of SA, such as the one proposed by Wells (106), a framework where maladaptive cognitions trigger anxiety and avoidance behaviors that reinforce dysfunctional beliefs. For these individuals, exercise settings are not neutral environments but are laden with perceived social threat. Upon entering such a setting, their negative core beliefs (“I am incompetent,” “I am physically inadequate”) are activated. This triggers a shift to intense self-focused attention and monitoring of their own physiological sensations (e.g., heart rate, sweating), which they catastrophically misinterpret as signs of imminent health failure or social humiliation. To prevent this feared outcome, they engage in “safety behaviors,” with the most definitive being complete avoidance of the activity. This avoidance, while reducing short-term anxiety, prevents any opportunity to disconfirm their negative beliefs, thus trapping them in a powerful, self-perpetuating cycle of inactivity and psychological distress.

### 5.4 Practical implications

Our findings offer several actionable implications for university administrators, public health planners, and campus wellness professionals. First and foremost, the identification of three distinct profiles underscores the need to tailor interventions. Rather than universal campaigns, universities should develop differentiated programs. For the large “Moderate-Adapting” group, low-barrier initiatives that build a supportive campus culture may be sufficient to “nudge” them toward more consistent activity. For the “Unhealthy-Anxious” group, however, more intensive support is needed, such as small, women-only fitness classes in private settings or workshops based on Cognitive Behavioral Therapy (CBT) to directly address social-evaluative fears related to exercise.

Second, these results advocate for the integration of mental and physical health services on campus. Our study shows that SA is a significant barrier to the health-promoting behavior of physical activity. Therefore, university wellness initiatives should embed SA screening into routine health assessments to identify at-risk students. This would facilitate early intervention and collaboration between counseling services and physical education departments to provide holistic support.

Third, institutions can leverage digital platforms to reach students who may be hesitant to seek face-to-face help. For students in the “Unhealthy-Anxious” profile, telehealth counseling, mental health apps focused on managing anxiety, or virtual fitness classes can serve as a less intimidating first step to engagement. These tools can help build self-efficacy in a private setting before transitioning to in-person activities.

Finally, our findings should inform policy and practice at an institutional level. The results provide a clear evidence base for allocating resources toward a multi-tiered system of support that addresses the specific psychological needs of different student profiles. By investing in targeted, evidence-based programs, universities can more effectively support student wellbeing, improve long-term health trajectories, and foster a campus environment where all students have the opportunity to thrive.

### 5.5 Strengths and limitations

This study has several strengths. First and foremost is its dual-analytic approach, which integrates both variable-centered (mediation) and person-centered (LPA) methods. This methodological synergy provides a more comprehensive understanding than either approach could alone. The mediation analysis illuminates how SRH relates to PA on average—identifying SA as a key mechanism—while the person-centered analysis reveals who is most affected by identifying distinct subgroups with different risk patterns. This moves beyond a ‘one-size-fits-all’ model and allows for the identification of a high-risk ‘Unhealthy-Anxious’ group that requires targeted intervention. Second, the study addresses a significant public health concern—physical inactivity—within a large and understudied population of female Chinese university students, providing valuable data to inform culturally specific health promotion efforts.

Despite the valuable insights provided, this study has several limitations that should be acknowledged.

First, the study employed a cross-sectional design, which captures data at a single point in time. Although our mediation model is based on self-presentation theory, the relationships between SRH, SA, and PA are likely to be complex and potentially bidirectional. For example, it is plausible that low levels of physical activity could lead to a decline in self-rated health and an increase in SA over time. Future research should utilize longitudinal or experimental designs to disentangle the causal pathways and temporal precedence among these variables.

Second, all primary variables were measured using self-report questionnaires. This method is susceptible to potential biases, such as social desirability bias (e.g., participants might over-report their physical activity levels or under-report their SA) and recall bias. Although self-report measures are common and practical for large-scale surveys, incorporating objective measures in future studies, such as using accelerometers to track physical activity or conducting clinical interviews to assess SA, would enhance the validity and reliability of the findings.

Third, the sample was limited to female college students from some universities in China. This specificity limits the generalizability of our findings to male students, students from other universities or cultural backgrounds, and young adults who are not in higher education. The unique academic and social pressures faced by this demographic may influence the observed relationships, and future research is needed to test whether these profiles and associations hold true in more diverse populations.

Fourth, and relatedly, physical activity was assessed with a single-item measure. While this approach is efficient for large-scale screening, a single item cannot capture the multifaceted nature of physical activity, including its various dimensions like frequency, duration, intensity, and type (e.g., aerobic, strength training, leisure-time). This may lead to an oversimplification of individuals’ activity levels and a less precise estimation of the relationship between physical activity and other variables. Future research should consider employing more comprehensive, validated multi-item questionnaires, such as the International Physical Activity Questionnaire (IPAQ), to obtain a more nuanced assessment.

Finally, while we controlled for several demographic variables, there may be other unmeasured confounding variables that could influence the results. Factors such as socioeconomic status, depression, academic major, personality traits (e.g., neuroticism, extraversion), and access to recreational facilities could also play a role in shaping students’ health perceptions, anxiety levels, and activity patterns. Future studies could benefit from including a broader range of potential covariates to provide a more comprehensive understanding of these complex relationships.

Collectively, these limitations mean our findings should be interpreted with caution. They provide a valuable snapshot and a strong theoretical model, but the magnitude of the effects may be biased, and the causal direction remains unconfirmed. The results are best understood as a foundational step highlighting a high-risk group and a key psychological mechanism that now require more robust, longitudinal, and multi-measure investigation.

## 6 Conclusion

This study adopted a dual-analytic approach to investigate the links between SRH, SA, and PA in female college students. The mediation analysis demonstrated that SA partially mediates the SRH–PA relationship, while the person-centered analysis revealed three distinct profiles with significantly different PA levels. Together, these findings highlight SA as a key psychological mechanism and underscore the heterogeneity within female student populations.

These results call for moving beyond one-size-fits-all strategies. Tailored interventions should be designed for each subgroup: sustaining intrinsic motivation in the “Healthy-Resilient,” providing supportive nudges for the “Moderate-Adapting,” and delivering CBT-informed, low-stigma programs for the “Unhealthy-Anxious.” Future research should employ longitudinal designs to establish causality, incorporate qualitative methods to understand subgroup experiences, and replicate findings across diverse cultural contexts.

At a policy level, these findings provide a foundation for evidence-based programming in university health systems. Embedding SA screening into routine campus health assessments, integrating mental health services with physical education programs, and leveraging digital platforms such as mobile apps and online counseling could create accessible, targeted solutions. Investing in such integrated approaches is not ancillary but essential to supporting student wellbeing and long-term public health.

## Data Availability

The original contributions presented in the study are included in the article/Supplementary material, further inquiries can be directed to the corresponding author.
