# Supplementary material for: Exploring self-rated health, physical activity, and social anxiety among female Chinese university students: a variable- and person-centered analysis
Source: Front Public Health. 2025 Sep 18;13:1681504. doi: 10.3389/fpubh.2025.1681504 (PMC12488654; doi:10.3389/fpubh.2025.1681504)
Supplement: Supplementary file 1 [file Data_Sheet_1.docx]

**Self-Perceived Health Scale**

This instrument comprises 12 items distributed across three sub-dimensions. Respondents indicated their level of agreement with each statement (e.g., “I can keep my balance, for example, by standing on one foot”) on a 6-point Likert scale,where 1 was “Disagree”, 2 “Slightly agree”, 3 “Agree”, 4 “Mostly agree”, 5 “Strongly agree”, and 6 “Absolutely agree”.The total score represents the level of Self-Rated Health.

|  | Item | Disagree | Slightly agree | Agree | Mostly agree | Strongly agree | Absolutely agree |
| --- | --- | --- | --- | --- | --- | --- | --- |
| 1 | I feel emotionally healthy. | 1 | 2 | 3 | 4 | 5 | 6 |
| 2 | I am in adequate emotional health. | 1 | 2 | 3 | 4 | 5 | 6 |
| 3 | I consider myself to be a person with emotional well-being. | 1 | 2 | 3 | 4 | 5 | 6 |
| 4 | I have adequate mental health. | 1 | 2 | 3 | 4 | 5 | 6 |
| 5 | I can walk easily. | 1 | 2 | 3 | 4 | 5 | 6 |
| 6 | I can move my limbs (arms, legs, and/or head) without effort. | 1 | 2 | 3 | 4 | 5 | 6 |
| 7 | I can keep my balance, for example, by standing on one foot. | 1 | 2 | 3 | 4 | 5 | 6 |
| 8 | I can go up and down stairs with ease. | 1 | 2 | 3 | 4 | 5 | 6 |
| 9 | My lifestyle helps me take care of my health. | 1 | 2 | 3 | 4 | 5 | 6 |
| 10 | My habits allow me to improve my health. | 1 | 2 | 3 | 4 | 5 | 6 |
| 11 | My diet allows me to maintain good health. | 1 | 2 | 3 | 4 | 5 | 6 |
| 12 | I feel that my daily activities improve my health. | 1 | 2 | 3 | 4 | 5 | 6 |

**Social Anxiety**

The SIAS-6 and SPS-6 scales along with the original statements in brackets. Items 1–6 are from the Social Interaction Anxiety Scale (SIAS), and items 7–12 are from the Social Phobia Scale (SPS).Both measures have 6-item scales, and each item is scored on a scale from 0 (“Not at all characteristic of me”) to 4 (“Extremely characteristic of me”). The total score of each item represents the level of social anxiety of the participants, and the higher the score, the higher the level of social anxiety assessment.

|  | Item | Not at all characteristic of me | A little  Characteristic of me | Much  Characteristic of me | Enough Characteristic of me | Extremely characteristic of me |
| --- | --- | --- | --- | --- | --- | --- |
| 1 | I have difficulty making eye contact with others. | 0 | 1 | 2 | 3 | 4 |
| 2 | I find it difficult mixing comfortably with the people I work with. | 0 | 1 | 2 | 3 | 4 |
| 3 | I tense up if I meet an acquaintance on the street. | 0 | 1 | 2 | 3 | 4 |
| 4 | I feel tense if I am alone with just one person. | 0 | 1 | 2 | 3 | 4 |
| 5 | I have difficulty talking with other people. | 0 | 1 | 2 | 3 | 4 |
| 6 | I find it difficult to disagree with another’s point of view. | 0 | 1 | 2 | 3 | 4 |
| 7 | I get nervous that people are staring at me as I walk down the street. | 0 | 1 | 2 | 3 | 4 |
| 8 | I worry about shaking or trembling when I’m watched by other people. | 0 | 1 | 2 | 3 | 4 |
| 9 | I would get tense if I had to sit facing other people on a bus or train. | 0 | 1 | 2 | 3 | 4 |
| 10 | I worry I might do something to attract the attention of other people. | 0 | 1 | 2 | 3 | 4 |
| 11 | When in an elevator, I am tense if people look at me. | 0 | 1 | 2 | 3 | 4 |
| 12 | I can feel conspicuous standing in a line. | 0 | 1 | 2 | 3 | 4 |
